# Targeting PD-L1 (Programmed death-ligand 1) and inhibiting the expression of IGF2BP2 (Insulin-like growth factor 2 mRNA-binding protein 2) affect the proliferation and apoptosis of hypopharyngeal carcinoma cells

**DOI:** 10.1080/21655979.2021.1983278

**Published:** 2021-10-05

**Authors:** Xudong Yang, Jisheng Liu

**Affiliations:** a Department of Otolaryngology, First Affiliated Hospital of Soochow University, Soochow, Jiangsu, P.R. China; b Department of Otolaryngology, Affiliated Hospital of Jiangsu University, Zhenjiang, Jiangsu, P.R. China

**Keywords:** Apoptosis, hypopharyngeal carcinoma, insulin-like growth factor 2 mRNA-binding protein 2, programmed cell death 1 ligand 1, proliferation

## Abstract

Programmed cell death-ligand 1 (PD-L1) have been attracting increasing attention in cancer diagnosis and treatment. The insulin-like growth factor 2 mRNA-binding protein 2 (IGF2BP2) is involved in the progression of multiple types of cancer. So, the role of IGF2BP2 and PD-L1 in hypopharyngeal carcinoma was assessed. Western blotting and immunochemistry were used to evaluate the expression of IGF2BP2 and PD-1/PD-L1. IGF2BP2 expression was knocked down in FaDu cells, and the effects on cell viability, apoptosis and proliferation were measured. A tumor-bearing nude model of hypopharyngeal carcinoma was constructed to evaluate the effect of a PD-L1 inhibitor and IGF2BP2 knockdown on hypopharyngeal carcinoma *in vivo*. RNA pull-down assays were used to assess the interaction between IGF2BP2 and PD-L1. The results showed that knockdown of IGF2BP2 inhibited FaDu cell proliferation and promoted apoptosis, as evidenced by the lower cell viability, a higher ratio of TUNEL-positive cells, decreased expression of Bcl-2 and cyclins, and increased expression of cleaved-caspase 3. *In vivo*, the tumor volume and weight were reduced by both the PD-L1 inhibitor and IGF2BP2 knockdown. Additionally, the interaction between PD-L1 and IGF2BP2 was confirmed. In conclusion, the results in the present study revealed that inhibition of IGF2BP2 might be a potentially relevant method for treating hypopharyngeal carcinoma, and the effects might be mediated via inhibition of the PD-1/PD-L1 axis.

## Introduction

Hypopharyngeal cancer is one of the most common types of cancer, accounting for 2–6% of neck and head cancers [1,2]. In recent years, the incidence rate of hypopharyngeal carcinoma has been increasing gradually, particularly in younger individuals [3]. Due to its unique anatomical structure, the clinical symptoms are atypical during the early stages, and once noticeable clinical symptoms manifest, the cancer has usually progressed. Hypopharyngeal carcinoma is usually accompanied by lymph node metastasis, which is easy to misdiagnose and is associated with a poor prognosis [4]. According to the location of the tumor, hypopharyngeal cancer can be divided into three types: Pyriform sinus carcinoma, posterior wall cancer and post cricoid carcinoma. The primary therapeutic approach to hypopharyngeal cancer includes surgery, radiotherapy and systemic chemotherapy [5–8]. As a highly malignant tumor, hypopharyngeal carcinoma is prone to cervical lymph node metastasis and distant metastasis. Thus, there is an increasing need to identify novel therapeutic target and develop drugs for the treatment of hypopharyngeal carcinoma.

Immunotherapy has become one of the most promising methods for the management of several types of cancer [9–13]. Tumor immunotherapy aims to mobilize the body’s immune system and enhance anti-tumor immunity, so as to suppress and kill tumor cells. Programmed cell death-1 (PD-1) was initially derived from tumor cell/T-cell hybrids. The receptor is present on the surface of T cells as well as primary B cells, and serves an important role in the differentiation and apoptosis of immune cells [14]. PD-1 has two ligands, PD-L1 (B7-H1) and PD-L2 (B7-DC), which belong to the B7 family of proteins [15]. PD-L1 is expressed in several human tumor tissues, and is able to effectively inhibit the activation, proliferation and secretion of cytokines related to T cells, the immune response and to induce T-cell failure or even apoptosis upon binding to PD-1. PD-1/PD-L1 inhibitors have achieved good therapeutic effects in non-small cell lung cancer, melanoma, advanced Hodgkin’s lymphoma, liver cancer and breast cancer, amongst other types of cancer [16–19].

Insulin-like growth factor 2 mRNA-binding protein 2 (IGF2BP2) is a member of the conserved single-stranded RNA binding protein family IGF2, and is expressed in a wide range of fetal tissues, as well as in >16 types of cancer, but only in a limited number of normal adults’ tissues [7]. IGF2BP2 can function as a post-transcriptional regulator of mRNA localization, stability and translation, and its dysregulated expression is usually associated with various types of cancer, such as colorectal, breast, pancreatic and non-small cell lung cancer [20–23]. Previous studies have shown that IGF2BP2 expression is increased in several types of carcinomas, and that it is associated with cancer cell proliferation, migration, adhesion and energy metabolism [20]. In the present study, the roles of IGF2BP2 and PD-1/PD-L1 in hypopharyngeal carcinoma were investigated.

## Materials and methods

### Patients

Patients diagnosed with squamous cell carcinoma from February 2019 to January 2020 were retrospectively screened. A total of 24 patients were included in this study and informed consent was obtained from all patients with the approval of the First Hospital of Soochow University (approval number: KY2019011506). All but one of the tumors were located in the pyriform sinus, with the remaining tumor being located in the posterior hypopharyngeal wall. The clinicopathological characteristics of these patients are shown in Table 1.

**Table 1.** The clinical data for the patient in this study

Gender	Age	Tumor location	Clinical stages	Histopathological type	pTNM
Male	51	Right pyriform sinus	T1N1M0	Squamous-cell carcinoma	– – -
Male	69	Left pyriform sinus	T2N1M0	Squamous-cell carcinoma	pT2N0M0
Male	59	Left pyriform sinus	T3N2M0	Squamous-cell carcinoma	pT3N2M0
Male	67	Left pyriform sinus	T4N1M0	Squamous-cell carcinoma	pT4N1M1
Male	55	Left pyriform sinus	T2N1M0	Squamous-cell carcinoma	– – –
Male	80	Right pyriform sinus	T4N2M0	Squamous-cell carcinoma	pT3N2M0
Male	41	Right pyriform sinus	T2N1M0	Squamous-cell carcinoma	pT2N0M0
Male	54	Left pyriform sinus	T2N0M0	Squamous-cell carcinoma	pT2N1M0
Male	70	Left pyriform sinus	T3N2M0	Squamous-cell carcinoma	pT3N2M0
Male	69	Left pyriform sinus	T3N0M0	Squamous-cell carcinoma	pT3N1M0
Male	67	Left pyriform sinus	T3N1M0	Squamous-cell carcinoma	pT3N1M0
Male	70	Left pyriform sinus	T4N2M0	Squamous-cell carcinoma	pT4N2M1
Male	62	Left pyriform sinus	T2N1M0	Squamous-cell carcinoma	pT2N1M0
Male	81	Left pyriform sinus	T1N2M0	Squamous-cell carcinoma	pT1N2M0
Male	81	Right pyriform sinus	T3N1M0	Squamous-cell carcinoma	pT3N1M0
Male	51	Right pyriform sinus	T2N1M0	Squamous-cell carcinoma	pT2N2M0
Male	63	Right pyriform sinus	T3N2M0	Squamous-cell carcinoma	pT3N2M0
Male	59	Right pyriform sinus	TIN0M0	Squamous-cell carcinoma	pT1N0M0
Male	82	Right pyriform sinus	T3N1M0	Squamous-cell carcinoma	pT3N1M0
Male	63	Right pyriform sinus	T2N1M0	Squamous-cell carcinoma	pT2N2M0
Male	68	Left pyriform sinus	T1N2M0	Squamous-cell carcinoma	pT1N2M1
Male	56	Right pyriform sinus	T2N1M0	Squamous-cell carcinoma	pT3N1M0
Male	48	Right pyriform sinus	T1N2M0	Squamous-cell carcinoma	pT1N2M0
Male	57	Posterior hypopharyngeal wall	T1N1M0	Squamous-cell carcinoma	pT1N1M0

### Cell culture and treatment

The normal human nasopharyngeal epithelial cell line NP69SV40T and pharyngeal carcinoma cell lines, including FaDu, PCI-12, HN-16 and BICR-6, were obtained from ATCC and cultured in RPMI-1640 medium supplemented with 10% FBS and 0.5% penicillin streptomycin l-glutamine mixture. Cells were cultured in a humidified incubator at 37°C with 5% CO_2_.

For IGF2BP2 knockdown, short hairpin RNA (shRNA) against IGF2BP2 and scrambled negative control (NC) vector were designed and synthesized by Shanghai GenePharma, Co., Ltd. For *in vitro* transfection, FaDu cells were transfected with shRNA-IGF2BP2 or shRNA-NC using Lipofectamine® 2000 (Invitrogen; Thermo Fisher Scientific, Inc.) according to the protocol of the manufacturer, and as described previously [24]. Briefly, lipofectamine 2000 was mixed with 20 μg plasmids, which was then added to cells and incubated at 37°C for 6 h. After 48 h transfection, the transfection efficacy was validated by RT-qPCR and successfully transfected cells were selected for subsequent experiments.

### Cell-counting kit-8 (CCK-8) assay

Cell viability was determined using a CCK-8 assay (Beyotime Institute of Biotechnology). Control or transfected cells were seeded in a 96 well plate at a density of 5 × 10^3^ cells/well and incubated for 12, 24 and 48 h at 37°C. Next, 10 μl CCK-8 solution was added, and the cells were cultured for a further 1.5 h at 37°C. The absorbance at 450 nm was measured using a microplate reader (Bio-Rad Laboratories, Inc.).

### Reverse transcription-quantitative (RT-q) PCR

Total RNA from FaDu cells was extracted using TRIzol® reagent (Invitrogen; Thermo Fisher Scientific, Inc.) and concentrations were measured using a NanoDrop system (R&D Systems, Inc.). Total RNA (2 μg) was reverse-transcribed into cDNA using a PrimeScript™ RT reagent kit (Takara Bio, Inc.). qPCR was performed using 1 μl cDNA per well, TaqMan MasterMix (Applied Biosystems) and 250 nM (final concentration) each of the sense and antisense primers. The PCR reaction procedure was as follows: 5 min at 95°C, with 40 cycles of 30 sec at 95°C and 45 sec at 65°C. The sequences of the primers used for qPCR were: IGF2BP2 forward, 5ʹ-CGGGGAAGAGACGGATGATG-3ʹ and reverse, 5ʹ- GGTAGTCCACGAAGGCGTAG-3ʹ; GAPDH forward, 5ʹ-GAAAGCCTGCCGG TGACTAA-3ʹ and reverse, 5ʹ-GCCCAATACGACCAAATCAGAGA-3ʹ. Results were evaluated using the 2^−∆∆Cq^ method [25] and the calculated number of copies was normalized to that of GAPDH mRNA in the same sample.

### TUNEL staining

Apoptotic FaDu cells were visualized using TUNEL staining according to the manufacturer’s protocol (Nanjing KeyGen Biotech Co., Ltd.). Briefly, cells that were cultured on cover slips were fixed using 4% neutral buffered formalin solution at room temperature for 25 min. The nuclear was stained with DAPI (Nanjing KeyGen Biotech Co., Ltd.) at room temperature in the dark for 10 min. And then images were captured in 3 random fields using a fluorescence microscope.

### Western blotting

Total protein was extracted from cultured FaDu cells using RIPA lysis buffer (Thermo Fisher Scientific, Inc.). The lysed cells were centrifuged and the protein concentration was determined using a Bio-Rad protein assay. The total proteins (40 μg) were subjected to 15% SDS-PAGE for separation. The resolved proteins were transferred to PVDF membranes, which were blocked in fresh 5% nonfat milk at room temperature for 2 h. Then, the membranes were incubated with the primary antibody at 4°C overnight, followed by incubation with the secondary antibody at room temperature for 2 h. An ECL kit (Thermo Fisher Scientific, Inc.) was used to visualize signals. Protein expression levels were semi-quantified by Image-Pro Plus software version 6.0 (Roper Technologies, Inc.).

### Immunohistochemical analysis

Immunohistochemical staining was performed to evaluate the expression of PD-L1. The tissues were cancerous and paraneoplastic tissues from patients with hypopharyngeal cancer, and were fixed in 4% paraformaldehyde at 4°C for 12 h. After dehydrating using a graded series of ethanol solutions and cleared using xylene, the samples were embedded in paraffin. After the sections (5 μm thick) of hypopharyngeal tissue were deparaffinized, rehydrated and blocked with 10% goat serum at room temperature for 2 h, they were incubated with the primary antibody (anti-PD-L1; Abcam, ab205921; 1:500) overnight at 4°C and secondary antibody (goat anti-rabbit secondary antibody; Abcam, ab6721; 1:1000) at room temperature for 10 min in succession. Subsequently, tissues were counterstained using hematoxylin and observed under a light microscope (Carl Zeiss AG) and photographed using a digital camera (AxioCam MRc5; Zeiss AG). PD-L1 expression was shown by a brown or dark brown stain, while negative expression was indicated by blue staining. The darker the brown color, the higher the expression.

### Nude mouse model of hypopharyngeal carcinoma and treatment

All animal studies were approved by the Animal Studies Ethics Committees of the First Affiliated Hospital of Soochow University (approval no. IACUC-20190022-8). The FaDu human pharyngeal squamous carcinoma cells were grown to 80% cell confluence and trypsinized (0.25% trypsin). The cells were washed with PBS, centrifuged, collected, and resuspended in 0.9% NaCl solution. A total of 20 (5 per group) six-week-old male BALB/C nude mice (20–25 g weight) were anesthetized by i.p. injection of 40 mg/kg pentobarbital, and then subcutaneously underarm injected with 0.1 ml (1.5 × 10^6^) FaDu cell suspension to establish the hypopharyngeal carcinoma model. A swab was used to prevent cancer cells from spilling and bleeding. The entire process was performed under strict sterile conditions. In the PD-L1 inhibitor treatment group, the mice were treated with 200 μg Pidilizumab (dissolved in sterile saline). The body weight of mice and tumor volumes were measured every 3 days. A total of 21 days after the first injection, mice were euthanized by intraperitoneal injection of 200 mg/kg pentobarbital, and mice exhibited no spontaneous breathing and no blink reflex within 2–3 minutes were considered as death. The tumors were immediately removed and weighed. Tumor volumes were calculated using the following equation: Tumor volume (mm^3^) = length (mm) × width (mm)^2^/2. The volume of the tumors was <1,000 mm^3^.

### RNA pull-down assay

For determining the interaction between PD-L1 RNA and IGF2BP2 protein, the FaDu cells were lysed with RIPA lysis buffer (Thermo Fisher Scientific, Inc.) and then incubated with biotinylated PD-L1 probes (Shanghai GenePharma, Co., Ltd.) at 4°C for 2 h, which was pre-bound to streptavidin magnetic beads (100 pmol of RNA per 50 μL magnetic beads; Invitrogen; Thermo Fisher Scientific, Inc.) at 4°C for 30 min. The mixtures were washed with 1x wash buffer and elution buffer (Thermo Fisher Scientific, Inc.), being centrifuged at 8000 × g and 4°C for 15 min and the supernatant was subjected to western blotting to analyze the binding proteins in the pull-down products.

### Statistical analysis

The experiments were repeated at least three times unless otherwise specified. Data are presented as the mean ± standard deviation (SD). GraphPad Prism (GraphPad Software, Inc.) was used to analyze the data. Differences between the means of the groups were compared using a paired Student’s t-test or a one-way ANOVA followed by Tukey′s test.

## Results

### PD-1/PD-L1 and IGF2BP2 expression is increased in hypopharyngeal carcinoma

**Figure 1.**
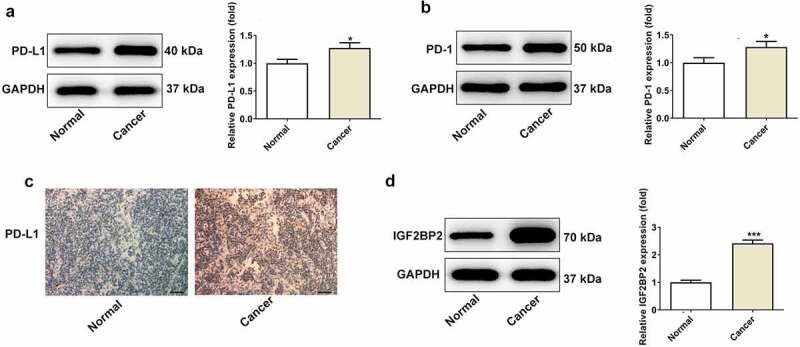
The expression of PD-L1, PD-1 and IGF2BP2 in pyriform sinus of healthy individuals and patients with hypopharyngeal carcinoma. The protein expression of PD-L1 (a) and PD-1 (b). (c) The immumohistochemical staining for PD-L1 (scale bar, 40 μm). (d) The protein expression of IGF2BP2 in normal and cancer tissues. *P < 0.05 and ***P < 0.001 vs normal group

The protein expression levels of PD-1/PD-L1 and IGF2BP2 was measured by western blotting. As shown in Figure 1, the protein expression levels of PD-L1 (Figure 1(a)), PD-1 (Figure 1(b)) and IGF2BP2 (Figure 1(d)) were increased in the hypopharyngeal cancer tissues. Consistently, immunohistochemical analysis showed that the expression of PD-L1 was increased in the hypopharyngeal cancer tissues (Figure 1(c)). Additionally, the protein expression levels of IGF2BP2 were also higher in the hypopharyngeal cancer tissues. Taken together, these results confirmed that PD-1/PD-L1 and IGF2BP2 expression is increased in hypopharyngeal cancer, and may serve as a therapeutic target for management of hypopharyngeal cancer.

### Knockdown of IGF2BP2 promotes apoptosis and inhibits proliferation of hypopharyngeal carcinoma cells

**Figure 2.**
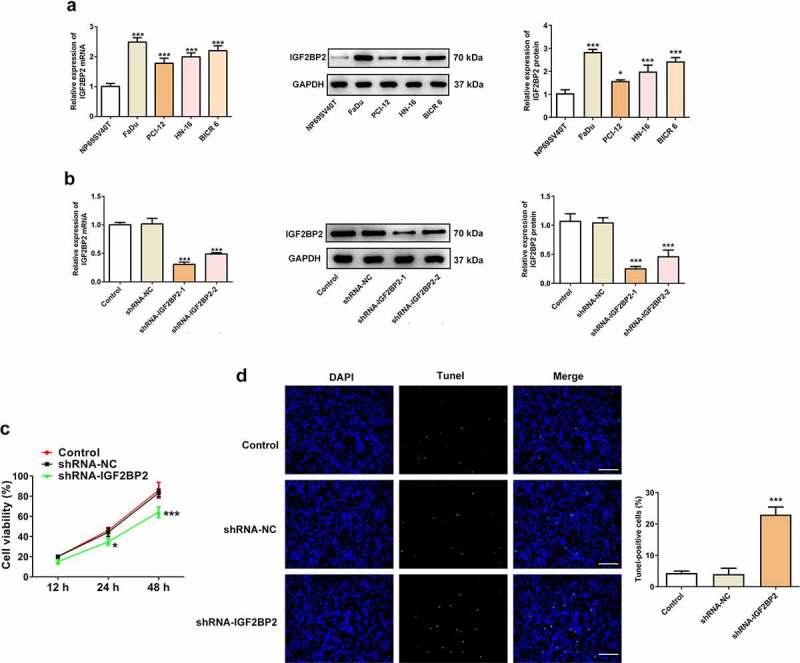
The effects of IGF2BP2 knockdown on cell viability and apoptosis. (a) The mRNA and protein expressions of IGF2BP2 in various hypopharyngeal carcinoma cells relative to normal cells NP69SV40T. *P < 0.05 and ***P < 0.001 vs NP69SV40T. (b) The mRNA and protein expressions of IGF2BP2 in FaDu cells or cells that transfected with shRNA-NC or shRNA-IGF2BP2. ***P < 0.001 vs control. (c) The cell viability of FaDu cells or cells that transfected with shRNA-NC or shRNA-IGF2BP2 at different times. *P < 0.05 and ***P < 0.001 vs shRNA-NC. (d) TUNEL staining images of FaDu cells or cells that transfected with shRNA-NC or shRNA-IGF2BP2. The nucleus was stained with blue and apoptotic cells were stained with green (scale bar, 50 μm)

**Figure 3.**
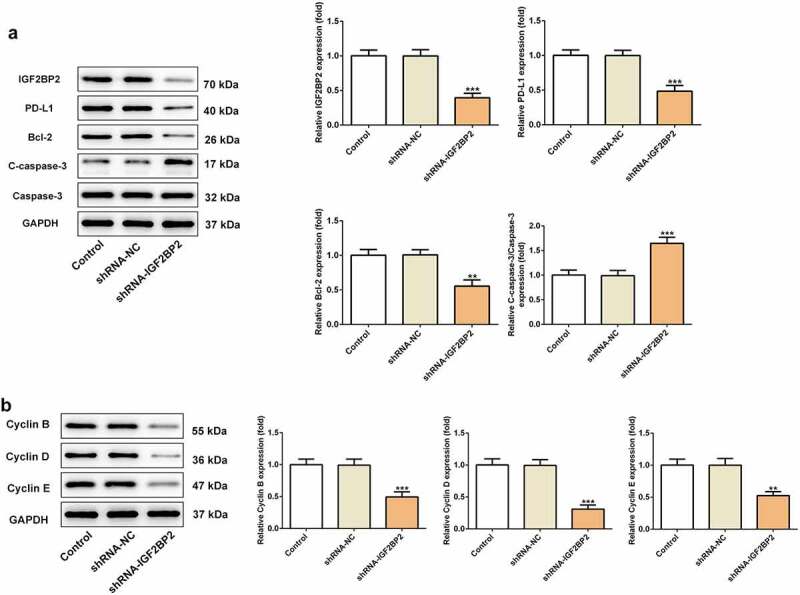
The effects of IGF2BP2 knockdown on proteins involved in apoptosis and cell cycle. (a) the protein expression of IGF2BP2, PD-L1, Bcl-2 and cleaved-caspase 3 (C-caspase-3). (b) the protein expression of cyclin B, D and E in control FaDu cells or cells that transfected with shRNA-NC or shRNA-IGF2BP2. **P < 0.01 and ***P < 0.001 vs shRNA-NC

IGF2BP2 expression was significantly upregulated in hypopharyngeal carcinoma cell lines compared with that in the normal human nasopharyngeal epithelial cell line, NP69SV40T (Figure 2(a)). FaDu cells were used for all subsequent experiments, as they exhibited the highest level of expression of IGF2BP2. IGF2BP2 expression was knocked down by shRNAs. shRNA-IGF2BP2-1 was used for subsequent experiments, because it exhibited the greatest knockdown efficiency (Figure 2(b)). As shown in Figure 2(c), the cell viability was significantly reduced in the cells transfected with shRNA-IGF2BP2. Additionally, TUNEL staining showed that knockdown of IGF2BP2 enhanced the ratio of apoptotic cells (Figure 2(d). Knockdown of IGF2BP2 decreased expression of the anti-apoptotic protein Bcl-2, whereas expression of the pro-apoptotic protein, cleaved-caspase 3, was increased (Figure 3(a)). Knockdown of IGF2BP2 also reduced the expression of PD-L1 (Figure 3(a)). The expression of cell cycle-related proteins, including cyclins B, D and E were all decreased by IGF2BP2 knockdown, indicating the cell cycle progression was arrested when IGF2BP2 expression was decreased (Figure 3(b)).

### Knockdown of IGF2BP2 and inhibition of PD-L1 reduces the growth of hypopharyngeal cell xenograft tumors in nude mice

**Figure 4.**
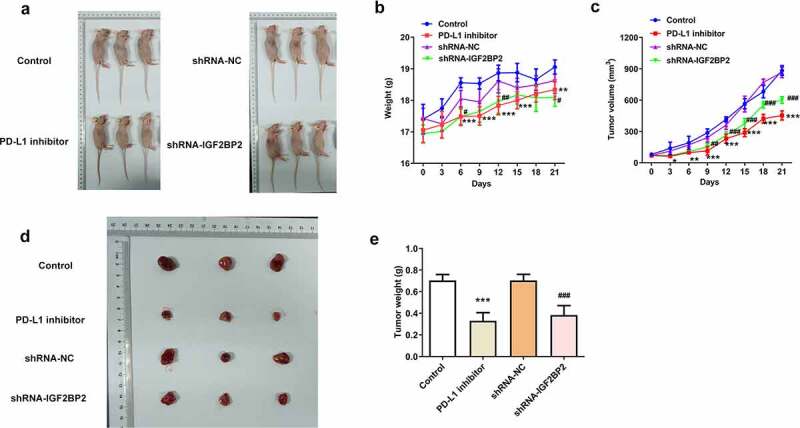
The effects of PD-L1 inhibitor and IGF2BP2 knockdown on hypopharyngeal carcinoma tumor growth in xenograft nude mice. (a) Representative images of xenograft nude mice in different groups. The body weight (b) and tumor volumes (c) of mice in different groups at different days post transplantation. Representative images (d) and weight (e) of tumors in different groups at 21-day post transplantation. *P < 0.05, **P < 0.01 and ***P < 0.001 vs control; ^#^P < 0.05, ^##^P < 0.01 and ^###^P < 0.001 vs shRNA-NC

To further confirm the role of IGF2BP2 in hypopharyngeal carcinoma *in vivo*, normal FaDu cells or cells transfected with shRNA-IGF2BP2 or shRNA-NC were transplanted into mice. For PD-L1 inhibition, normal FaDu xenograft mice were treated with the PD-L1 inhibitor pidilizumab. As shown in Figure 4(a–c), in the group treated with the PD-L1 inhibitor or transplanted with the shRNA-IGF2BP2 cells, body weight and tumor volume was relatively lower than the control group or shRNA-NC group, respectively. The final tumor weight of mice after sacrificing was significantly lower in the group treated with PD-L1 inhibitor or in mice transplanted with the shRNA-IGF2BP2 cells compared with the control group (Figure 4(d,e). Taken together, the results demonstrated that inhibition of IGF2BP2 or PD-L1 expression reduced tumor growth.

### Reciprocal regulatory association between IGF2BP2 and PD-L1 expression

**Figure 5.**
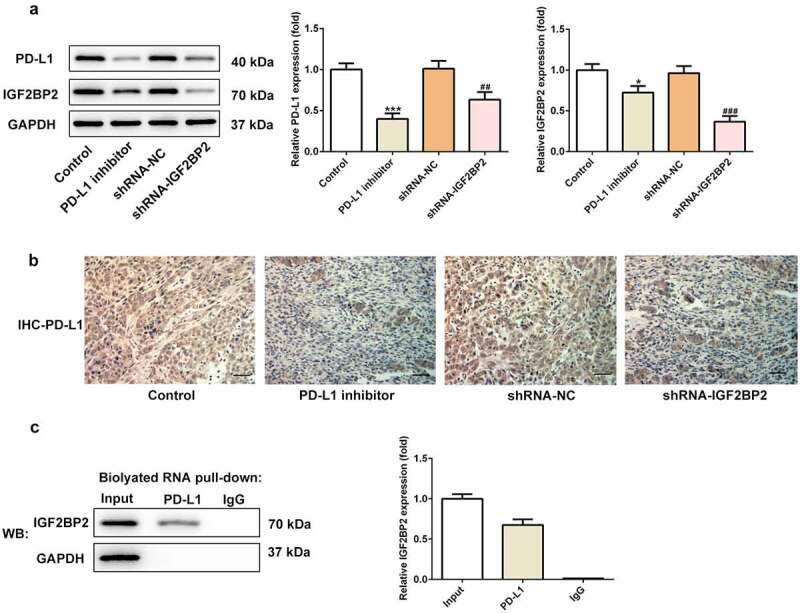
The relationship between IGF2BP2 and PD-L1. (a) the protein expression of PD-L1 and IGF2BP2 in tumor tissues of nude mice in different groups. (b) the immumohistochemical staining for PD-L1 in tumor tissues of nude mice in different groups (scale bar, 40 μm). (c) RNA pull-down assay was utilized to detect the interaction between IGF2BP2 and PD-L1 RNA. *P < 0.05 and ***P < 0.001 vs control; ^##^P < 0.01 vs shRNA-NC

As shown in Figure 5(a), the expression of PD-L1 in the groups treated with the PD-L1 inhibitor or transfected with shRNA-IGF2BP2 was decreased compared with the control group. Additionally, the expression of IGF2BP2 was also reduced in the group treated with the PD-L1 inhibitor. Immunohistochemical analysis was performed to further confirm these results (Figure 5(b)). The above results suggested that IGF2BP2 and PD-L1 may interact directly and reciprocally regulate each other’s expression. Consistent with this hypothesis, the results of the RNA-pull down assay verified the presence of a direct interaction between IGF2BP2 and PD-L1 (Figure 5(c).

## Discussion

PD-L1 is expressed in immune and tumor cells, and has become a topic of intense interest [26,27]. PD-L1 is reported to be associated with the prognosis of several diseases. PD-L1 levels also serve as a noninvasive biomarker that can be used to diagnose head and neck cancer [28,29]. PD-L1 expression differs across different tissues, individuals and diseases. High expression of PD-1 and PD-L1 indicate a comparatively good prognosis in ovarian cancer [30]. By contrast, high PD-L1 expression in non-small cell lung cancer was indicative of recurrence following surgery compared with the patients with low PD-L1 expression [31]. Co-expression of PD-L1 and PD-1 is associated with an improved prognosis in patients with gastric cancer [32]. The levels of PD-1 and PD-L1 have also been shown to vary based on surgery and the length of treatment; thus, they may represent valuable biomarkers that may be used to monitor the status of the cancer.

In the present study, the effects of PD-L1/PD-1 in hypopharyngeal carcinoma were investigated. In order to avoid false positive results, both western blotting and immunochemistry were used to evaluate the expression of PD-L1/PD-1. The results showed that the expression of both PD-L1 and PD-1 was elevated in the tissues of patients with hypopharyngeal carcinoma, indicating that PD-L1 together with PD-1 may serve as potential biomarkers for treating hypopharyngeal carcinoma, and inhibition of PD-L1 may be a novel therapeutic approach to the management of hypopharyngeal carcinoma. Pidilizumab is an inhibitor of PD-L1, and was used in the present study. In a nude mouse model of hypopharyngeal carcinoma, the expression levels of PD-L1 and PD-1 were further evaluated using western blotting and immunochemistry. In the model group and PD-L1 inhibitor treatment group, the tumor volumes and weights in the mice were lower in the PD-L1 inhibitor group compared with the model group, suggesting that PD-L1 inhibitors suppressed tumor growth in a nude mouse model of hypopharyngeal carcinoma. Western blotting and immunochemical analysis showed that PD-L1 and PD-1 expression was decreased in the PD-L1 inhibitor treatment group compared with the model group.

As an mRNA-binding protein, IGF2BP2 is widely known to function as an essential oncogene via targeting lncRNAs and miRNAs, thereby promoting cancer cell invasion, proliferation and migration [33–35]. For example, IGF2BP2 was reported to be stabilized by lncRNA LINRIS, in turn promoting colorectal cancer [20]. In addition, IGF2BP2 was regulated by CCN6 protein in metaplastic carcinomas of the breast [22]. In this study, it was shown that IGF2BP2 expression was also elevated in the tissues of patients with hypopharyngeal carcinoma, indicating its potential role in hypopharyngeal carcinoma. Therefore, IGF2BP2 expression was knocked down in FaDu cells, and this resulted in reduced cell proliferation and increased apoptosis, suggesting its inhibitory effect on hypopharyngeal carcinoma. The results from the *in vivo* studies were consistent with those of the *in vitro* experiments, demonstrating that both inhibition of PD-L1 and knockdown of IGF2BP2 could significantly reduce the growth of hypopharyngeal tumors.

Furthermore, knockdown of IGF2BP2 reduced PD-1/PD-L1 expression, and the presence of the PD-L1 inhibitor could decrease IGF2BP2 expression. Thus, it was hypothesized that IGF2BP2 may bind to PD-L1 mRNA, affecting PD-L1 and PD-1 protein expression, thereby serving a role in the progression of hypopharyngeal neoplasms. The results from RNA pull-down assays confirmed the binding between PD-L1 mRNA and IGF2BP2 protein. Therefore, as with PD-L1 inhibition, IGF2BP2 knockdown may inhibit PD-1 expression via binding to PD-L1 mRNA, ultimately reducing the progression of hypopharyngeal neoplasms. However, how PD-L1 inhibition reduced IGF2BP2 expression remains unknown. It is hypothesized that IGF2BP2 could bind to PD-L1 mRNA to enhance PD-L1 protein expression, and PD-L1 would in turn induce IGF2BP2 expression. However, whether this feedback loop exists and the specific mechanism involved remain to be determined.

## Conclusion

The findings of the present study demonstrated that IGF2BP2 together with PD-1/PD-L1 may serve as effective biomarkers for the diagnosis and prognosis of hypopharyngeal carcinoma. Furthermore, inhibition of IGF2BP2 and/or PD-L1 function may be a novel approach to the management of hypopharyngeal carcinoma.

## Data Availability

All datasets used or analysed during the current study are included in this manuscipt.

## Highlights

(1) Knockdown of IGF2BP2 promoted apoptosis and inhibited proliferation of hypopharyngeal carcinoma cells.

(2) Knockdown of IGF2BP2 and inhibition of PD-L1 reduced hypopharyngeal cell xenograft tumor growth in nude mice.

(3) IGF2BP2 and PD-L1 might regulate each other.
